# Institutional Epistemic Isolation in Psychiatric Healthcare

**DOI:** 10.1080/02691728.2024.2403620

**Published:** 2024-09-30

**Authors:** Lucienne Jeannette Spencer

**Affiliations:** Department of Psychiatry, University of Oxford, Oxford, UK

**Keywords:** Epistemic injustice, gatekeeping, mental health, institutional injustice

## Abstract

Within the last decade, epistemic injustice has been a valuable framework for those working on exposing oppressive practices within the healthcare system. As this work has evolved, new terminology has been added to the epistemic injustice literature to bring to light previously obscured epistemic harms in healthcare practices. This paper aims to explore an important concept that has not received the attention it deserves: epistemic isolation. By developing Ian Kidd and Havi Carel’s concept of epistemic isolation, a new range of epistemic harms are brought to the fore, as some of the most marginalised in our society are forced to operate from positions of ignorance. In the words of Kidd and Carel, epistemic isolation occurs in ‘situations where a person or group lacks the knowledge of, or means of access to, particular information; for instance, if they live within a politically repressive society which forbids access to the necessary sources of information in order to protect the government’s hegemony’ (183–184). This paper will demonstrate that such epistemic isolation is uniquely devastating for those with psychiatric illness, exacerbating their already challenging circumstances.

## 1. Introduction

Within the last decade, epistemic injustice has been a valuable framework for those working on exposing oppressive practices within the healthcare system. As this work has evolved, new terminology has been added to the epistemic injustice literature to bring to light previously obscured epistemic harms in healthcare practices. This paper aims to explore an important concept that has not received the attention it deserves: epistemic isolation. By developing Ian Kidd and Havi Carel’s concept of epistemic isolation, a new range of epistemic harms are brought to the fore, as some of the most marginalised in our society are forced to operate from positions of ignorance. In the words of Kidd and Carel, epistemic isolation occurs in ‘situations where a person or group lacks the knowledge of, or means of access to, particular information; for instance, if they live within a politically repressive society which forbids access to the necessary sources of information in order to protect the government’s hegemony’ (Kidd and Carel 2017, 183–184). This paper will demonstrate that such epistemic isolation is uniquely devastating for those with psychiatric illness, exacerbating their already challenging circumstances.

## 2. Hermeneutical Injustice in Psychiatric Healthcare

Hermeneutical injustice occurs when there are gaps in the interpretive framework where the experiences of marginalised groups ought to be (Fricker 2007).^1^ This can occur through ‘unequal hermeneutical participation’ in the meaning-making process or what Fricker terms ‘hermeneutical marginalisation’ (Fricker 2007, 152). But when certain groups are excluded from the construction of interpretive frameworks, fundamental interpretations that are significant to these groups are left as gaps in the hermeneutical resources. Fricker refers to these as ‘hermeneutical lacunas’ (Fricker 2007, 151). Consequently, hermeneutical marginalisation triggers hermeneutical injustice, whereby gaps in the collective understanding obscure the experiences of marginalised groups. Fricker understands hermeneutical resources as essential for meaning-making; where hermeneutical resources are absent from the interpretive framework, the subject’s grasp of their experience is distorted, limited or otherwise confined.

Unlike testimonial injustice, Fricker understands hermeneutical injustice as a ‘somewhat indirect’ discrimination because ‘the injustice will tend to persist regardless of individual efforts’ (Fricker 2017, 54). Fricker does not suggest here that individuals experience hermeneutical injustice indirectly. Rather, hermeneutical injustice is grounded in *structural* hermeneutical marginalisation as opposed to one individual directly enacting epistemic harm on another. The injustice lies in the wider social structure, as certain groups are excluded from contributing to a shared interpretative framework. Accordingly, hermeneutical injustice typically endures despite the hearers’ attempts to understand the speaker, as the interpretive framework renders the marginalised speaker almost unintelligible. Nevertheless, José Medina clarifies that the agent’s responsibility is not diminished in the case of hermeneutical injustice. As Medina points out, there is collective culpability for hermeneutical injustice as ‘an entire culture can be held responsible for not trying to understand a particular kind of experience or a particular kind of subjectivity’ (Medina 2017, 42). Medina claims ‘we can identify degrees of complicity in how individuals respond to lacunas and limitations in the hermeneutical resources they have inherited and in how they participate (or fail to participate) in expressive and interpretive dynamics’ (Medina 2017, 42–43).

Communication difficulties are a natural barrier in the psychiatric encounter, as the experiences of patients are profoundly difficult to put into words (Spencer and Kidd 2023). Healthcare professionals attempted to overcome the inevitable communication barriers posed by psychiatry through the development of a universal psychiatric vocabulary. This universal vocabulary took the form of diagnostic manuals, the most popular today being the *Diagnostic and Statistical Manual of Mental Disorders* (DSM). According to the APA, the aim of the latest edition of the DSM is as follows:

[to create] a common language for clinicians to communicate about their patients and [establish] consistent and reliable diagnoses that can be used in the research of mental disorders. It also provides a common language for researchers to study the criteria for potential future revisions and to aid in the development of medications and other interventions. (APA, 2013)

Whilst the DSM may go some way towards creating a universal framework for understanding psychiatric illness amongst healthcare professionals and researchers, what is missing from this mission statement is the pursuit of a common language between clinician and *patient*. By emphasising communication *about* patients rather than *to* patients, the American Psychiatric Association (APA) suggests that the patient’s understanding of their psychiatric illness is secondary to that of the clinician and researcher.

Thus, despite the central role the doctor-patient dialogue plays in psychiatric healthcare, the voice of the person with psychiatric illness is notably lacking in diagnostic manuals. This constitutes a hermeneutical marginalisation. An epistemic privilege is afforded to the third-person perspective of the DSM and the psychiatrist who wields it: ‘the medical perspective is regarded not only as authoritative but often even exclusive of other perspectives, such that medical diagnosis effectively constitutes a monopoly on the way the experience is interpreted’ (Scrutton 2017, 349). As a result, the sense-making of psychiatric illness has been limited to that which can be conveyed through the language of the DSM.

Numerous harms emerge from such structural hermeneutical injustice. The primary harm is the product of epistemic injustice more broadly, as it captures the harm of being undermined as an epistemic subject: ‘they are wrongfully excluded from participation in the practice that defines the very core of the very concept of knowledge’ (Fricker 2007, 145). As rational agents, undermining one’s capacity to give knowledge is to undermine something central to being human: ‘when someone suffers a testimonial injustice, they are degraded *qua* knower, and they are symbolically degraded *qua* human’ (Fricker 2007, 44). Explicitly, it is not merely the individual who suffers a deflated epistemic status but the marginalised social group that the individual seemingly represents.

This primary harm can also be understood as compromising one’s epistemic agency; epistemic agency can be attributed to a person ‘when we believe the person has the capacity to produce and share knowledge competently and authoritatively’ (Houlders, Bortolotti, and Broome 2021, 7690). This perceived epistemic agency is fundamental to one’s sense of humanity. For Fricker, to undermine a person’s capacity ‘to know’ is to undermine an aspect of a person that makes them distinctly human. It is this distortion of a person’s humanity that makes the primary harm *intrinsic*. In a case of hermeneutical injustice, the primary harm takes the form of a ‘situated hermeneutical inequality’, whereby knowers are disadvantaged by gaps in the interpretative framework (Fricker 2007, 7).

The primary harm of hermeneutical injustice is captured in Fricker’s paradigmatic example of sexual harassment. Carmita Wood’s status as a ‘knower’ was relegated, as she was robbed of invaluable hermeneutical resources to describe her experience of sexual harassment. Her capacity as a ‘knower’ was inhibited as she could not make sense of her experience. Fricker understands such a primary harm to be a profound assault upon the constitution of the subject. Her concern is how we ‘receive the word of others’ and how this can impact the other’s very sense of self (Fricker 2007, 168). The intrinsic nature of this primary harm derives from its ability to ‘go more or less deep in the psychology of the subject […] where it goes deep, it can cramp self-development, so that a person may be, quite literally, prevented from becoming who they are’ (Fricker 2007, 5). As such, the subject undergoes what Fricker terms a ‘cognitive disadvantage’ as they cannot fully comprehend (or interpret) an area of their experience. This cognitive disadvantage’s disabling effect is apparent in the ‘life-changing flash of enlightenment’ experienced when a hermeneutical lacuna has been overcome (Fricker 2007, 153). Fricker illustrates an instance of ‘hermeneutical breakthrough’ via the case of Wendy Sanford, who is introduced to the term ‘postpartum depression’ after participating in a university-based workshop. In a ‘life-changing forty-five minutes’, she can make sense of her own experience of postpartum depression. Consequently, a ‘hermeneutical darkness’ is ‘suddenly lifted from Wendy Sandford’s mind’ (Fricker 2007, 149). The epiphany that occurs through the creation of hermeneutical resources demonstrates the debilitating effect of hermeneutical injustice.^2^

## 3. Epistemic Isolation

In the last 60 years, an eruption of activism has developed new and vital ways of understanding psychiatric illness outside the sanist^3^ standards that governed them previously. Groups such as the ‘psychiatric survivors’ movement’ that emerged in the late 1960s and the ‘neurodiversity’ movement of the 1990s called for people with psychiatric illness to be given the power to define themselves in their own terms. For instance, proponents of the neurodiversity movement champion terminology such as ‘those with neurocognitive differences’ and ‘neurominorities’ in place of ‘those with mental disorders’ or ‘mental illness’, as they consider the latter to reinforce pathological models. In addition, the term ‘neuro-typical’ is opted for in place of ‘normal’ or ‘sane’ (Chapman 2019). Such pathbreaking activism inspired a wealth of academic research, dubbed ‘mad studies’ by Richard Ingram (Ingram 2007). An abundance of pathographies emerged that described the experiences of psychiatric illness and the healthcare system in their own words, casting aside the interpretive framework instilled by a sanist society.

With such an abundance of first-person hermeneutical resources, it may seem strange to suggest that someone with a psychiatric illness may be confronted by a hermeneutical lacuna. Yet, many people with psychiatric illness are subjected to what Kidd and Carel call ‘epistemic isolation’: the ill person is socially positioned in such a way that they do not have access to this wealth of hermeneutical resources (Kidd and Carel 2017). Consequently, there are countless reports of people who were left unaware that what they were experiencing was a psychiatric illness for most of their lives. Such epistemic isolation constitutes the ‘situated hermeneutical inequality’ that renders some people unable to access the appropriate hermeneutical resources to describe their experience (Fricker 2007, 162). Thus, epistemic isolation is distinct from hermeneutical lacunae in the following way: a wealth of hermeneutical resources exists; however, marginalised groups may be forced into a position of ignorance, isolated from these essential resources. In what follows, I shall consider the forms epistemic isolation may take in psychiatric healthcare.

First, epistemic isolation may occur if the person has an illness that is not well-known or particularly rare where the hermeneutical resources to describe the experience are less numerous and are therefore less likely to be encountered. In fact, Fricker provides an example of just such epistemic isolation through the previously discussed case of postpartum depression. Fricker recounts Wendy Sandford discovering the term ‘postpartum depression’ (a type of mood disorder experienced after childbirth) following her participation in a workshop with other women with the condition (Brownmiller 1990, 182). While the term already existed, Wendy Sandford was epistemically isolated in such a way that she did not have access to the notion of postpartum depression. Epistemic isolation of this form occurs through a lack of ‘familiarity’ with a given illness, to use Corrigan and colleagues’ sense of the word (Corrigan et al. 2003, 166). Corrigan and colleagues refer to familiarity as the knowledge we possess of an illness (their focus is on mental illness, but familiarity could also extend to somatic illnesses): ‘it varies in intensity from viewing television portrayals of mental illness, to having a friend or co-worker with mental illness, to having a family member with mental illness, to having a mental illness oneself’ (Corrigan et al. 2003, 166). If there is a lack of familiarity with an illness, say if postpartum depression is rarely talked publicly about or portrayed in the media, people with the illness are likely to be isolated from important hermeneutical resources. This lack of familiarity seems to disproportionately occur in cases where marginalised groups face illnesses, as their experiences are occluded from public understanding.

Second, epistemic isolation may occur when an illness is so skewed in the public understanding that those with the condition may never assume that they have it. For example, someone may suppose that they cannot have autism because they’re nothing like the lead in *Rain Man*, that they cannot have PTSD because they’re not a soldier or that they cannot have OCD because they are not obsessed with cleaning (Scriver 2019; Spencer and Carel 2021). In this case, a person may be epistemically isolated from a proper understanding of a condition due to the trivialisation or distortion of the illness. Perhaps most simply of all, the ill person may have been brought up with sanist ideologies that distort their perception of psychiatric illness:

I was raised to not believe in psychiatric help. I was raised to believe mental illness didn’t really exist. I was raised to believe taking pills for mental illness just showed weakness. I was raised to believe that all these drugs were just a ‘big pharma’ conspiracy. (Tracy 2016, 15)

Third, someone may be epistemically isolated if they are socially positioned in such a way that they do not have access to insightful pathographies. This is all the more likely considering the strong correlation between low income and illness and therefore exclusion from vital resources. The ill person may belong to a socio-demographic where illness is unlikely to be discussed. This may be particularly common in cases of psychiatric illness. For example, men, older people and African Americans are the least likely groups to seek psychiatric diagnosis (Affleck, Carmichael, and Whitley 2018; Campbell and Mowbray 2016; Leong and Zachar 1999).

Various harms may emerge from the three forms of epistemic isolation discussed above. Perhaps most profoundly, those who are epistemically isolated are put at a ‘cognitive disadvantage’ as they are blocked from comprehending an essential aspect of their lived experience. Such cognitive disadvantage can be particularly detrimental to those with psychiatric illnesses, whose sense of self is often particularly vulnerable. Examples of the impact of epistemic isolation can be found in the literature on female autism: as autism is perceived to be a male condition, autistic females are less likely to receive an autism diagnosis. As one neurodiverse woman, diagnosed at 35, expressed: ‘I felt different and I felt there was something wrong with me. And obviously there isn’t. But because I didn’t know I was autistic, I felt like I was wrong and I didn’t fit in. That’s a horrible feeling to have for 30-plus years’ (National Autistic Society, n.d.). Thus, epistemic isolation may impact one’s capacity for self-understanding and epistemic agency. An epistemically isolated individual is unable to produce and share knowledge in an authoritative manner.

The fourth form of epistemic isolation is particularly insidious and will serve as the focus for the remainder of this paper. This form of epistemic isolation occurs after diagnosis and within the healthcare system. As such, I recognise this form of epistemic isolation as institutional. One may assume that a person with psychiatric illness would no longer be epistemically isolated once they are ushered into the healthcare system. Unfortunately, this is not always the case. Building on Kidd and Carel, I argue that one way in which hermeneutical lacunas can emerge is through an imposed epistemic isolation by the healthcare professional. Kidd and Carel describe epistemic isolation as ‘situations where a person or group lacks the knowledge of or means of access to, particular information’ (Kidd and Carel 2017, 183–184). In the medical context, the patient may have epistemic isolation imposed upon them if the healthcare professional withholds vital information concerning the patient’s treatment plan, the nature of their illness or even the very diagnosis itself. From such epistemic isolation, hermeneutical lacunas emerge in the patient’s understanding of their illness. The focus will be on how this epistemic isolation occurs within psychiatric healthcare. I identify three different kinds of information the psychiatric patient can be obstructed from through institutionalised epistemic isolation: the treatment plan, the meaning of their diagnosis and (or) the diagnosis itself.

## 4. Institutionalised Epistemic Isolation

Though it is no longer standard practice, it was once common for medical practitioners to withhold a diagnosis of life-threatening somatic illnesses from their patients. This practice was widespread in cases of a cancer diagnosis. As cancer was once considered terminal, some medical practitioners called into question whether the disclosure of a cancer diagnosis constituted a harm inflicted upon the patient. Arguments against disclosure portrayed the diagnosis as an unnecessary, anxiety-inducing burden on the patient. Indeed, cancer was often only referred to as ‘the c-word’, as if the word ‘cancer’ were harmful in itself. Yet, as Susan Sontag observed, it is not the word ‘cancer’ that produces a detrimental effect for the patient but the social understanding of cancer as an ‘invincible predator’ (Sontag 2002, 7). Consequently, she proposed that ‘the solution is hardly to stop telling the cancer patient the truth, but to rectify the conception of disease, to de-mythicize it’ (Sontag 2002, 7).

Following improved survival rates and revised therapeutic practices available to patients diagnosed with life-threatening illnesses, there was a significant shift in the policy and practice of disclosure in Western medicine by the late 1970s (Sokol 2006). Medical professionals are now more or less unanimously in favour of the patient’s right to disclosure in somatic illness, not least because of the development of patient rights and increased litigation (Dégi 2009).^4^ Nevertheless, in the domain of psychiatric illness, the debate about the risks and benefits of diagnosis disclosure rages on.

Psychiatric healthcare professionals possess knowledge and expertise in the objective aspects of psychiatric illness through extensive training in this field. As such, they have the authority to disseminate knowledge regarding the patient’s diagnosis and treatment. This constitutes an epistemic privilege. By virtue of this authority, they possess a unique power to grant or conceal medical information. According to a literature review, the disclosure of psychiatric diagnosis has increased from 30–65% pre-2000s to 77–88% post-2000s (Milton and Mullan, 2014: 263). The persistence of non-disclosure practices (12–23%) stems from a conflict between the patient’s ‘right to know’ their diagnosis and the ‘non-maleficence principle’, which dictates that the healthcare professional must avoid further harm to the patient (Carpiniello and Wasserman 2020, 3). Analogous to the arguments put forward against the disclosure of cancer diagnoses, it is argued that the patient’s ‘right to know’ may be waived in favour of the principle of ‘therapeutic privilege’, according to which ‘the physician feels obliged to forego full disclosure, in order to safeguard the patient’s wellbeing’ (Carpiniello and Wasserman 2020, 3). It could be argued that non-disclosure is preferable for the following reasons: 1) the diagnosis itself is stress-inducing, 2) it carries a high level of stigmatisation, 3) the patient has a tendency towards negative emotional behaviour and 4) there is uncertainty regarding the validity of the diagnosis. The most common undisclosed psychiatric illnesses are schizophrenia (Milton and Mullan, 2014, 266) and borderline personality disorder (Lequesne and Hersh 2004).

The impact of non-disclosure practices in psychiatric healthcare is uncovered through service-user reports. For instance, Fenton et al. revealed that all but one of the six participants in their study reported a lack of explanation from the healthcare professionals regarding their condition and how treatment would proceed (Fenton et al. 2014). In cases where the diagnosis is withheld from the patient, they often experience a deflation of their credibility status: ‘they hadn’t actually given me a diagnosis. They were never really straight with me or explained to me what the problem was, so I just thought they probably thought I was an attention-seeker’ (Bonnington and Rose 2014, 13).^5^

Despite these ongoing debates in the field of psychiatric care, the healthcare professional’s responsibility to disclose a psychiatric diagnosis is surprisingly absent from the numerous and lengthy guidelines on ethical practices in psychiatric healthcare (Royal College of Psychiatrists: Confidentiality and Information Sharing 2017; General Medical Council 2020; Reforming the Mental Health Act; 2021). While it is easy to find guidelines on the ethics involved in diagnosis disclosure to third parties (such as family members, carers and employers) in psychiatric healthcare, I have been unable to find ethical guidelines concerning disclosure to the patient. In addition, there is little research to support the argument that diagnosis disclosure constitutes a harm in itself. In fact, there is growing research that supports the positive impact of disclosure. In 2017, Blessing et al. conducted a study to determine the risks and benefits of disclosing a psychosis diagnosis to the patient. The study found that ‘after disclosure of diagnosis, all individuals reported less psychological distress’ and ‘all individuals seem to benefit from disclosure of diagnosis on a symptom level’ (Blessing et al. 2017, 3). Moreover, the study found that patients who were identified with an ‘at risk mental state’ have ‘a stronger belief that they can control events affecting them after disclosure of diagnosis’ (Blessing et al. 2017, 3). This study suggests that disclosure of diagnosis is more likely to improve the patient’s likelihood of recovery or at least the chances of ameliorating the effects of their illness.

Those studies that did find a negative impact upon the patient following the disclosure of diagnosis attributed the negative impact to the stigmatisation attached to the diagnosis itself. Gallagher et al.’s study of patients’ reaction to receiving a diagnosis found that if the diagnosis is considered ‘bad news’, it is because ‘it is stigmatizing for participants and, it is suggested, for participants’ families, resulting in their not sharing the news or sanitizing what they share with others’ (cited in Gallagher et al. 2010, 38). For instance, upon receiving a diagnosis of bipolar disorder, one patient described feeling ‘as though I didn’t have a future, it was so shocking as to know what was going to happen. I had no idea how it was going to affect my life’ (cited in Gallagher et al. 2010, 38). This fear of stigmatisation seems to be further amplified by a lack of explanation from the healthcare professional about what bipolar disorder is and how it will be treated. In line with Sontag, I suggest that the negative impact here is not the disclosure of diagnosis, as if the word itself possesses a ‘magic power’ (Sontag 2002, 6). Rather, the negative impact ought to be attributed to the sanist attitudes attached to the diagnosis, plus a lack of clarification from the healthcare professional. In other words, the harm the healthcare professional fears and attempts to ameliorate is the result of prejudicial attitudes towards psychiatric illness, not the diagnosis itself. These sanist attitudes are then only further perpetuated by the secrecy surrounding the patient’s diagnosis, ‘implying that it is too terrible to tell the patient and too awful to discuss’ (Atkinson 1989, 24). Thus, it is the stigmatisation of psychiatric illness that needs to be tackled, not the act of diagnosis.

The problem of non-disclosure in psychiatric healthcare is part of a broader issue of withholding information from the patient, thus placing the psychiatric patient in a position of epistemic ignorance. As discussed above, while many clinicians do inform patients of their diagnosis, patients are often left in the dark as to what this diagnosis actually *means*. They may have been given a label, but the term ‘bipolar disorder’ or ‘schizophrenia’ is of little use if the patient is not made aware of the condition’s diagnostic features. This lack of understanding was revealed in the aforementioned study by Gallagher et al., where patients commonly reported an absence of explanation of their diagnosis from the healthcare professional. One patient said of her diagnosis of borderline personality disorder:

I didn’t get so much information of what it actually meant […] I think that sort of covered things like self-harming, in part, acting without thinking. I think the diagnosis that covered was basics, like ways that I behave and that was a surprise as well because I hadn’t heard of it, I didn’t understand what it was […]. (cited in Gallagher et al. 2010, 37)

Another patient recalls being told by her healthcare practitioner, ‘the good news is you don’t need to take medication anymore, the bad news is you’ve got a personality disorder so you no longer have ‘bi-polar’; she adds ‘so I actually hadn’t a clue what this meant’ (cited in Gallagher et al. 2010, 37).

Further studies have identified cases in which information regarding the *treatment plan* is withheld from psychiatric patients. This seems to be especially prevalent in those institutionalised under the Mental Health Act. In a study by Fenton et al., one patient reported his confusion over the medication he was receiving: ‘you don’t know what’s going on [laughing]. I must have been on quite a few different things … , it just seemed like they give me everything you know, or they tried to’ (Fenton et al. 2014, 236). Patients describe having to be insistent in their questioning of staff to access withheld information about their illness. One patient ‘described how any attempts ‘to try and find out more about it […] it was almost as though I had to be quite challenging to professionals, by being persistent’ (Horn, Johnstone, and Brooke 2007, 261). Patients who *did not* bombard the healthcare professionals with questions, ‘those who were quiet and posed no challenge’, were ‘more likely to get lost within the system’ (Wright et al. 2016, 372).

Some studies revealed a culture of service users placing the onus of combatting epistemic isolation on their fellow patients; they suggested that less demanding patients ought to speak up and ‘needed to be persistent and constantly ask for information’ if they wanted to gain access to the information they require (Wright et al. 2016, 372). However, routine testimonial injustice (let alone the person’s health condition itself) may make patients reluctant to approach the healthcare professional for information. They may fear (not unreasonably) that pressing for information may further perpetuate the credibility deficit attributed to them. As one patient observed, it is best to avoid persistent questioning, otherwise the patient would be ‘put under the hat of being a difficult client […] which as it turned out kind of reinforced the label for them’ (Horn, Johnstone, and Brooke 2007, 261).

I have identified three different kinds of information the psychiatric patient can be obstructed from through institutionalised epistemic isolation: the treatment plan, the meaning of their diagnosis and (or) the diagnosis itself. Epistemic isolation may occur accidentally due to the overwhelming work pressures of healthcare staff, leading them to deprioritise the epistemic needs of patients. Alternatively, it may be implemented intentionally, seemingly for the greater good of the patient. Either way, the healthcare professional does not intend harm to their patient; in fact, they often have the patient’s best interests at heart. Much institutional epistemic isolation is due to conformity to non-disclosure cultures in the psychiatric healthcare system.^6^ In the words of Kristie Dotson ‘epistemic injustice is very easy to commit. In fact, it is extraordinarily difficult to avoid it’ (Dotson 2012, 37).

Nevertheless, such epistemic isolation maintains the unequal credibility economy in the psychiatric healthcare system, as it places the patient at an epistemic disadvantage from the outset. Institutionalised epistemic isolation produces numerous harms. For one, it exacerbates the power imbalance between patient and healthcare professional and inhibits the development of a trusting, respectful therapeutic relationship. Moreover, much like the previously discussed forms of epistemic isolation, it threatens the epistemic agency of the patient and puts the patient at a cognitive disadvantage. Through institutionalised epistemic isolation, the patient is robbed of the capacity to communicate effectively and to understand their condition coherently. Essentially, this is epistemic gatekeeping.

## 5. Epistemic Injustice or Epistemic Disadvantage?

Before drawing this paper to a close, there is a final question to answer: is the described institutionalised epistemic isolation really an epistemic *injustice*? Drawing on Kidd and Carel, Rena Goldstein develops a rich account of epistemic isolation in her paper ‘Epistemic Disadvantage’ where she distinguishes between epistemic isolation as an epistemic *injustice* and as an epistemic *disadvantage* (Goldstein 2022). In line with Fricker, Goldstein recognises that not all epistemic harms are necessarily epistemic injustices. Indeed, as Goldstein observes, Fricker refers to certain epistemic harms as resulting from ‘circumstantial epistemic bad luck’ as opposed to epistemic injustice (Fricker 2007, 152). The example Fricker provides for this is, in fact, one from a medical context, as she imagines an illness that goes undiagnosed because knowledge of that illness does not yet exist (Fricker 2007, 152). While Fricker limits ‘epistemic bad luck’ to the product of ‘hermeneutical disadvantage’, Goldstein rightly broadens this notion to encompass a variety of epistemic harms that are not unjust but rather accidental in nature. Goldstein refers to these forms of epistemic harm as emerging from ‘Epistemic Disadvantage’: ‘epistemic disadvantage marks when a person or group is warranted excluded from knowledge exchanges, but the exclusion results in an intellectual or moral harm’ (Goldstein 2022, 1862).

Epistemic isolation is one such epistemic harm that can result from either an epistemic injustice or an epistemic disadvantage. Goldstein argues epistemic isolation is an epistemic disadvantage when it meets the following criteria:

1. Harms are non-deliberate, arising in circumstances of bad luck.
2. Speakers lack precision or mastery of concepts to effectively communicate their experience.
3. Affected participants are justifiably excluded or subordinated from the practice that could make the concept known. (Goldstein 2022, 1870–1871)

Goldstein’s aim of teasing apart ‘unjust’ from ‘accidental’ epistemic harms is an important one; it is essential that we avoid diluting the definition of epistemic injustice to the point that the term loses its meaning by ushering in epistemic harms that are not unjust. Therefore, we must understand whether the form of institutionalised epistemic isolation I have proposed in these pages is a form of epistemic injustice or an epistemic disadvantage.

However, before I evaluate institutionalised epistemic isolation against Goldstein’s criteria, I propose a modification. I propose that we revise Goldstein’s first criteria for epistemic disadvantage, as Fricker insists that epistemic injustice is necessarily non-deliberative. Therefore, being non-deliberate is not a distinguishing criterion for epistemic disadvantage. In 2017, Fricker sought to uncover how the concept of epistemic injustice had evolved over the decade since its conception and which features she recognises as absolute. The ‘absence of deliberate, conscious manipulation’ was the key feature that Fricker insisted is essential for epistemic injustice (Fricker 2017, 54):

I was trying to bring out a phenomenon that is easy to miss, and in need of a name. In this kind of epistemic injustice, the hearer makes a special kind of misjudgement of the speaker’s credibility - one actually clouded by prejudice. And this is importantly different from any deliberate misrepresentation of someone’s true or reasonable beliefs as false or rationally unfounded … (Fricker 2017, 54)

While Fricker acknowledges the urgency of drawing attention to intentional epistemic harms, the epistemic injustices she conceives of are ‘discriminatory but ingenuous misjudgement, and it will … be useful to continue keeping this separate from the closely related kind of injustice that involves the deliberate manipulation of others’ judgements of credibility’ (Fricker 2017, 54).

Of particular relevance to our discussion here is Fricker’s conception of hermeneutical injustice. Fricker understands hermeneutical injustice as a ‘somewhat indirect’ discrimination because ‘the injustice will tend to persist regardless of individual efforts’ (Fricker 2017, 54). In other words, it is grounded in structural hermeneutical marginalisation. The injustice lies in the wider social structure, as certain groups are excluded from contributing to a shared interpretative framework. Accordingly, hermeneutical injustice typically endures despite the hearers’ attempts to understand the speaker, as the interpretive framework renders the marginalised speaker almost unintelligible. In the case of sexual harassment, the marginalisation of the victims is built into the very structure of the interaction and has a scope that extends beyond the given interaction.

Following Kidd and Carel, I propose that epistemic isolation ought to be considered necessarily non-deliberative, regardless of whether it is an epistemic disadvantage or an epistemic injustice. To be clear, what is non-deliberative is the harm imposed on the epistemically isolated person. While a clinician may intentionally withhold hermeneutical resources from a patient, what is unintentional is the epistemic harm this leads to. Nevertheless, in line with Fricker, the fact that this epistemic harm is unintentional does not make it any less unjust.

In the medical context, a clinician would rarely intend to cause epistemic harm to their patient. Most clinicians intend the best outcomes for their patients, and any epistemic harm that may occur would likely be despite the clinician’s best efforts. However, in line with Fricker’s conception of epistemic injustice, the non-deliberate nature of this epistemic isolation does not make it any less unjust. As Goldstein rightly observes, epistemic injustice must be motivated by an identity prejudice. The implicit identity prejudice we can recognise here is that of pathophobia or ‘sanism’, as the patient is assumed to be unable to handle the information of their diagnosis or treatment plan, or it is assumed that it would not be properly understood even if carefully explained.^7^ Alternatively, epistemic isolation may occur because the epistemic needs of the patient (ensuring they understanding their diagnosis and treatment plan) are deprioritised in favour of other tasks, thus downgrading the patient’s epistemic agency. Although unintentional, psychiatric patients are deprived of essential information.

On the surface, it may appear that the latter, unintentional case would be an instance of ‘epistemic bad luck’ and does not involve identity prejudice due to its accidental nature. However, it would be wise to heed Dotson’s call to be suspicious of ‘epistemic bad luck’ (Dotson 2012). To refer to this as an instance of ‘epistemic bad luck’ would be to assume that it is merely circumstantial that the healthcare professional does not have time to meet the patient’s epistemic needs: to ensure they understand their treatment and diagnosis. The patient is, supposedly, just unlucky. However, this so-called ‘bad luck’ is driven by what Fricker calls an ‘institutional epistemic vice’ in the healthcare system (Fricker 2021).

Fricker defines institutional epistemic vice as ‘displayed – either in thinking or, where persistent, also at the level of institutional character – whenever there are culpable lapses in the institution’s epistemic ethos and/or in the implementation of its ends’ (Fricker 2021, 101). Fricker provides the example of a school, which has a good ethos and good epistemic commitments but has over time developed ‘sloppy information sharing’ (Fricker 2021, 100). So too, I argue that the psychiatric healthcare system has a good ethos, yet flawed information-sharing practices and an institutional attitude that deprioritises the epistemic needs of the patient. There is an institutional bias towards the epistemic needs of the healthcare professional over the epistemic needs of the patient. Consequently, the patient is isolated from pertinent knowledge.

Again, this does not mean that the healthcare professional does not wish they had the time to ensure the patient has the required hermeneutical resources. It is the institutional structure itself that deprioritises the epistemic needs of the patient, not the healthcare professional. This institutional epistemic isolation emerges from a structural identity prejudice which is not located in the individual healthcare professionals but rather exists in the bones of the institution. Therefore, epistemic isolation can be simultaneously non-deliberative and unjust. On this basis, I suggest that the first criteria ought to be adjusted to read ‘harms are not a result of identity prejudice and arise in circumstances of bad luck’. As argued above, the institutional epistemic isolation described in this paper results from identity prejudice rather than epistemic bad luck. With this established, let us now evaluate institutionalised epistemic isolation according to Goldstein’s latter two criteria.

Goldstein’s second criterion for epistemic disadvantage is as follows: ‘speakers lack precision or mastery of concepts to effectively communicate their experience’ (Goldstein 2022, 1870). This criterion emerges from Goldstein’s acknowledgement that in some spheres, there are experts and non-experts, and while this may cause a power imbalance, it is not necessarily an unjust one. This is indeed an important criterion. However, it can be a difficult one to evaluate. We need to be able to determine if an inability to effectively communicate one’s experience emerges from a lack of mastery or from an unjust interpretive framework. The first example that may come to mind could be a child’s lack of mastery over language to communicate their experiences effectively. However, this may not be a good example of a lack of mastery; as Carel and Györffy (2014) argue, the communicative abilities of children are often underestimated and wrongfully disregarded: ‘[the child’s] interpretative frameworks are at risk of rejection by adults, who, with few exceptions, cease to readily understand the child’s world’ (Carel and Györffy 2014, 1256).

Moving to the realm of psychiatry, it becomes arguably even harder to determine if communication difficulties result from the psychiatric illness itself or are due to an unjust interpretive framework. People with psychiatric illness often report an inability to effectively put their experiences into words. In *Darkness Visible*, for example, Styron describes depression as ‘so mysteriously painful and elusive in the way it becomes known to the self … as to verge close to being beyond description. It thus remains nearly incomprehensible to those who have not experienced it in its extreme mode’ (Styron [1990] 2010, 5). A difficulty in communicating one’s experience may be the result of the illness itself, as the experience is so beyond one’s conventional mode of existence that it defies articulation.^8^ However, as I have addressed in the first section of this paper, difficulties in communicating one’s illness experience may result from unjustly imposed hermeneutical frameworks. A ‘lack of precision or mastery of concepts’ may simply be a lack of precision or mastery in the imposed interpretive framework, in this instance a clinical one. One may have a good capacity for communicating their experience in their own words, beyond a clinical framework; however, such communication is not taken seriously. Often, it is likely to be a combination of both a natural and unnatural barrier to communication occurring simultaneously.

Rather than focusing on communicative abilities, it seems best to focus on whether there is a just division in epistemic labour between experts and non-experts. Goldstein follows Goldman (2001) in defining an expert as those with a ‘superior quantity or level of knowledge in some domain and an ability to generate new knowledge in that domain’ (Goldman 2001, 91). Clinicians fit this definition, as those who have developed skills in their field in diagnosing and treating illness and who act as generators of knowledge. However, those with psychiatric illness hold a unique form of knowledge and expertise; illness ‘gives us experiences that we would not otherwise have had and that we cannot know what it is like to have until we undergo them – knowledge that cannot otherwise be acquired’ (Carel et al. 2016, 1152). These can be understood as ‘transformative experiences’: certain experiences, can only truly be understood by those who have had both ‘the requisite bodily experience’ and, or, the requisite interpretation of the world (Kidd and Carel 2017, 185).

Although they may lack the clinician’s expertise in research skills, methodologies, diagnosis and treatment, the patient possesses experiential expertise in their given condition. They know how it feels to have the psychiatric illness in question. Therefore, if we take the ‘domain’ to be the psychiatric illness under evaluation, psychiatric patients cannot be said to lack a ‘superior quantity or level of knowledge’ or ‘an ability to generate new knowledge’. If the patient is treated as a non-expert from the outset, and the diagnosis or treatment plan is withheld, they are deprived of the opportunity to ‘master the concepts to effectively communicate their experience’ (Goldstein 2022, 1870). Instead, ignorance is imposed upon the patient from the offset. Moreover, while the patient may not have a mastery of the clinical terminology, the clinician should look for less exclusionary means of meaning-making with the patient. They may be able to fluently communicate their experience in their own words. In this sense, I argue that institutionalised epistemic isolation is an injustice rather than a disadvantage.

Finally, let us turn to the third criterion: ‘affected participants are justifiably excluded or subordinated from the practice that could make the concept known’ (Goldstein 2022, 1870–1871). As we have seen, clinicians and clinical institutions could attempt to justify withholding information through the ‘non-maleficence principle’, the idea that revealing a diagnosis or treatment plan will cause harm to the patient. Alternatively, it could be argued that the deprioritisation of the patient’s epistemic needs is justified due to limits on time and resources. However, through this paper, I have sought to dispel these assumptions, arguing that institutionalised epistemic isolation is not justified and is therefore epistemically unjust. I have demonstrated that withholding information from a patient often causes the patient more harm than good. Most significantly, institutionalised epistemic isolation places the patient at a ‘cognitive disadvantage’. As such, they are inhibited not only from contributing to the pool of knowledge regarding their illness but also from making sense of their illness themselves. This leads to what Fricker identifies as a primary epistemic harm: ‘they are wrongfully excluded from participation in the practice that defines the very core of the very concept of knowledge’ (Fricker 2007, 145). Although the diagnosis may be withheld due to the fear of stigmatisation, I have argued that it is the stigmatisation that causes harm, not the diagnosis itself. This stigmatisation is only exacerbated by a culture of silence around the diagnosis.

Goldstein provides a valuable development of epistemic isolation, and I share her concern for overstretching the term epistemic injustice beyond the unjust. I agree that certain people who experience an epistemic harm are, in fact, epistemically disadvantaged rather than subjected to an epistemic injustice, and this is an important distinction to make. I hope to have shown in this paper that institutionalised epistemic isolation is not such a case. Regarding Goldstein’s first criterion, I propose a shift in focus to whether or not an act of epistemic isolation is motivated by identity prejudice as opposed to whether it is deliberate; this is in line with Fricker’s understanding that an epistemic injustice can be both unjust and non-deliberate. Institutionalised epistemic isolation is indeed motivated by identity prejudice, as sanist attitudes either deprioritise patients receiving information or suggest that they are too fragile, or ‘too far gone’, to be able to handle it. Regarding Goldstein’s second criterion, I argue patients hold an experiential expertise which should be acknowledged as a valuable contribution to the medical domain. Although they may not be fluent in clinical jargon, they may be able to express their experiences in their own words that surpass the limits of clinical frameworks. In such a case of institutional epistemic isolation, the patient is often not given the opportunity to even attempt to master the language or make sense of their experience, as they are robbed of the basic information of their diagnosis and or treatment plan. Finally, as institutional epistemic isolation causes cognitive disadvantage, the supposed virtue of withholding information cannot be justified.

## 6. Conclusion

This paper has aimed to contribute to the work of Kidd, Carel and Goldstein in developing an account of epistemic isolation. I hope to shed new light on this distinct epistemic injustice, particularly for those with psychiatric illness. This paper identifies four forms of epistemic isolation, focusing on the fourth form: institutionalised epistemic isolation. Although my attention here is on the psychiatric institution, institutionalised epistemic isolation may occur in various institutions: the education system, sports, the department of the police and so on. While institutionalised epistemic isolation may have a unique impact on those in psychiatric healthcare, in that it impacts their identity and treatment in a distinct way, I believe it may be equally destructive for those caught in these alternative institutions. I encourage future research into how institutional epistemic isolation may occur in these domains and whether or not in these contexts they are instances of injustice or disadvantage.

This paper has demonstrated that receiving information regarding one’s diagnosis and treatment is more than just desirable for the patient; it is essential to the patient’s sense of self and sense of agency. In the words of Atkinson: ‘when a psychiatrist withholds a diagnosis from a patient he is denying the patient knowledge about his condition’ (Atkinson 1989, 24). Through epistemic isolation, the ill person is obstructed from vital information. Without these essential resources required to communicate their experiences, the psychiatric patient is met with hermeneutical injustice. As such, the psychiatric patient is forced to operate as an epistemic agent from the position of an imposed ignorance, where they are deprived of resources that are essential for communication and self-understanding. Thus, epistemic isolation acts as a mediator of knowledge, both outside and within the psychiatric healthcare system. I encourage future work that seeks to make the role of epistemic isolation more visible so that its pervasiveness and power might be better understood.

## Notes

1. Fricker refers to this as the ‘central case of hermeneutical injustice’ (Fricker 2007, 156). The ‘central case’ is a systematic injustice, which involves extensive and repeated bouts of hermeneutical harm. This contrasts with incidental hermeneutical injustice, which occurs as a one-off hermeneutical harm (Fricker 2007, 156).

2. For more on hermeneutical injustice in psychiatric healthcare, see Ritunanno 2022.

3. Like racism or sexism, Michael L. Perlin popularised the term ‘sanism’ to draw attention to the discriminatory distinction between the ‘mad’ and the ‘sane’ (Perlin 1992).

4. It is worth reiterating here that my discussion is limited to Western medical practice. Non-disclosure of terminal illness is still widely practised in other areas, such as China. See (Wang et al. 2018).

5. See also (Plahouras et al. 2020).

6. The psychiatric healthcare system could also be understood to be ‘institutionally opaque’, to use Carel & Kidd’s terminology. See (Carel and Kidd 2021).

7. There may of course be other intersectional identity prejudices at play concerning one’s gender, race, socio-economic status, disability and so on.

8. For more on this argument, see (Spencer 2023b).
